# Green Triage: A Curtain in Front of Possible Serious Injuries

**DOI:** 10.3390/healthcare13212691

**Published:** 2025-10-24

**Authors:** Gülşen Akçay, Bedriye Müge Sönmez, Hacer Demirdelen, Emre Çakar, Mert Şahin, Murat Özdemir, Mehmet Emin Arayıcı

**Affiliations:** 1 Department of Emergency Medicine, Etlik City Hospital, 06170 Ankara, Turkey; hacerdemirhan@gmail.com (H.D.); dremrecakar01@gmail.com (E.Ç.); mmerttsahin@gmail.com (M.Ş.); 2 Department of Emergency Medicine, Faculty of Medicine, Dokuz Eylül University, 35340 İzmir, Turkey; mugesonmez06@yahoo.com; 3 Air Ambulance Unit, Ministry of Health, 06800 Ankara, Turkey; doktoraiki@gmail.com; 4 Department of Biostatistics, Faculty of Medicine, Dokuz Eylül University, 35340 İzmir, Turkey; mehmet.e.arayici@gmail.com

**Keywords:** green triage, earthquake, mass casualty triage, emergency department

## Abstract

**Objective:** The human body cannot withstand intense mechanical forces generated by an earthquake. The shaking effect of an earthquake, as the human body absorbs it, produces both visible and invisible injuries. Therefore, it is essential to provide accurate triage in the process of mass casualties. Hence, this study aims to characterize green triage patients of the 2023 Kahramanmaraş, Turkey, earthquake and emphasize the need to reconsider mass casualty triage. **Methods:** A retrospective cross-sectional study was conducted on green triage patients who did not receive first-aid medical attention and attended the emergency department (ED) of a tertiary care hospital. The development of crush syndrome (CS), the receipt of renal replacement therapy (RRT), and associated injuries were considered. **Results:** Among 295 individuals, CS occurred in 32.2%, and RRT was required in 7.1% of cases. In addition to the traditional markers of CS, non-trapped green triage patients developed both CS and required RRT, as well as sustained injuries. Whether trapped or not, CK levels emerged as a significant predictor of CS. **Conclusions:** Green triage patients may acquire CS even in the absence of obvious injuries or entrapment. Accurate triage can be life-saving even in the absence of obvious injuries.

## 1. Introduction

Two consecutive earthquakes with a maximum moment magnitude (Mw) of 7.8 struck southeastern Turkey on 6 February 2023, resulting in over 50,000 deaths and more than 107,000 injuries, which re-emphasized the unpredictable nature of earthquakes as natural disasters [1,2].

The human body cannot withstand intense mechanical forces generated by an earthquake [3], and the shaking effect of seismic activity absorbed by the human body produces both overt and subtle injuries to it. Among the survivors, there exists a subgroup of individuals who extricate themselves from the rubble, either attempting to save their family or awaiting assistance from others, all while neglecting their own medical needs due to the psychological impact of the catastrophe. These individuals may incur mild to severe injuries yet remain oblivious to their medical urgency or deliberately disregard their condition in such exceptional circumstances. Hence, to avoid overlooking such patients in catastrophic events, they should be accurately managed. In this case, correct triage must be activated to connect them with the appropriate diagnostic and treatment options, as well as to determine which patients or injured people require urgent medical attention [4].

Accurate triage in mass casualties needs to be performed as intended and correctly classify patients, which will lead to inefficient use of resources, delays in meeting patients’ individual needs, patient unhappiness, adverse outcomes, and even patient death in extreme circumstances [5]. Several triage systems have been developed and utilized in mass casualty incidents across different countries, including the Simple Triage and Rapid Treatment (START), Sort, Assess, Life-Saving Interventions, Treatment and/or Transport, Sacco Triage Method, Careflight, and Triage Sieve; still, there is no consensus on which one is the best, because most of them were validated by simulations [6].

In disaster triage, the presence of a walking victim suggests green and secondary triage [7]. However, patients triaged in this category in a major earthquake could be as serious as those triaged in yellow and could even die from crush syndrome (CS) and acute kidney injury (AKI) [8,9]. In large-scale disasters, such as the 2023 Kahramanmaraş earthquake, the overwhelming demand for resources and the presence of many unknowns lead to prioritizing patient transport over triage, which can create an undertriage risk in any triage system [10]. Hence, this study aims to characterize green triage patients of the 2023 Kahramanmaraş, Turkey, earthquake and emphasize the need to reconsider mass casualty triage.

## 2. Materials and Methods

### 2.1. Study Design

This retrospective cross-sectional study was conducted on green triage patients who did not receive first-aid medical attention and presented to the ED of a tertiary care hospital within 5 days after the earthquake, which occurred between 6 February 2023, and 10 February 2023

### 2.2. Study Population

#### Inclusion and Exclusion Criteria

Patients aged 18 years or older, who were survivors of the earthquake, had not received first-aid medical attention, were ambulatory, and presented to the ED within 5 days after the earthquake, were included in the study. Patients who attended the ED after the specified period, received medical attention, presented from any healthcare facility with their own transportation or healthcare transfer services, or had missing data were excluded from the study.

A priori power analysis was conducted using GPower* version 3.1.9.7 to determine the minimum required sample size for detecting a statistically significant difference with a two-tailed test. The following parameters were used: effect size *d* = 0.3, a type 1 error probability (α) = 0.05, and power (1 − β) = 0.80. According to the computation, the noncentrality parameter (δ) was 2.814, the critical t value was 1.9687, and the degrees of freedom (df) were 272. The analysis indicated that a total of 274 participants were required to achieve the desired statistical power (actual power = 0.8007). To account for potential non-response or attrition, we increased the total sample size by approximately 5%, targeting a total of around 290 participants.

### 2.3. Data Collection

Demographic characteristics (age, gender, comorbidities, and drugs used), laboratory results [serum hemoglobin, leukocytes, platelets, urea, creatinine, sodium, potassium, calcium, phosphorus, creatine kinase (CK), CK-MB, and blood gas (pH, bicarbonate, and base deficit) values], presence of AKI, presence of myoglobunuria, transportation route (road or airway), date of presentation to the ED, time spent under the rubble, vital findings at admission, development of CS and receiving renal replacement therapy (RRT), injury severity score (ISS) and revised trauma score (RTS) results, presence and type of organ injury, and clinical outcome were recorded on previously prepared data forms.

Currently, CS lacks established diagnostic criteria. Nevertheless, a CK result more than five times the upper limit of normal has been employed as a diagnostic criterion in a large number of studies. In most cases, CK levels begin to rise 2–12 h after a muscle injury and peak between 24 and 72 h later [11]. Therefore, patients were identified as having CS if they had increased levels of CK, serum potassium, phosphorus, and myoglobinuria in the presence of suspected traumatic rhabdomyolysis, resulting in systemic complications, particularly AKI, and experienced oligo-anuria (<400 mL/day) or needed dialysis for a minimum of one day. CS, along with other nephrological disorders that may require dialysis, was defined as AKI. Oliguria (urine output ≤ 400 mL/day), high blood urea nitrogen (BUN; >40 mg/dL), elevated urea levels (>85.6 mg/dL), high serum creatinine levels (>2.0 mg/dL), high uric acid levels (>8.0 mg/dL), high potassium levels (>6.0 mEq/L), high phosphorus levels (>8.0 mg/dL), and low calcium levels (<8.0 mg/dL) were considered indicative of other nephrological conditions [1,12,13].

The development of CS and the receipt of RRT in non-trapped patients were the primary outcomes, while associated injuries were the secondary outcomes.

### 2.4. Ethical Considerations

The study was approved (approval no. AEŞH-BADEK-2024-123) by the Institutional Review Board of the hospital. Anonymity was maintained, and no personally identifiable information (such as names, addresses, or email addresses) was collected during the study period. As the study was a retrospective design, a consent form was not received from the participants. Only the researchers had the right to access information through the hospital database. The corresponding author stored the original database, and the patient registration numbers were hidden.

### 2.5. Statistical Analysis

All statistical analyses were conducted using STATA (v.18, College Station, TX, USA) to evaluate the relationships between crush syndrome and various demographic, bio-chemical, and clinical parameters. Continuous variables were assessed for normality (Kolmogorov–Smirnov test), and those following a non-normal distribution were presented as the median and interquartile range (IQR). In contrast, normally distributed variables were expressed as mean ± standard deviation (SD). Categorical variables were summarized as frequencies and percentages. Comparisons between individuals with and without crush syndrome were performed using the Mann–Whitney U test for non-normally distributed continuous variables and the independent samples *t*-test for normally distributed data. The chi-square (χ^2^) test or Fisher’s exact test was applied for categorical variables based on expected cell counts. Univariable *p*-values were considered exploratory and interpreted cautiously, given the multiplicity of tests. A multivariable logistic regression analysis was conducted to determine independent risk factors for crush syndrome. The model included age, sex, duration under rubble, pulse, CK, CK-MB, platelet count, RRT, and injury sites (thorax, thoracic, lumbar, and neurovascular) as covariates. The backward stepwise method was applied to refine the model. Odds ratios (ORs) with 95% confidence intervals (CIs) were reported for each predictor. Given the numerous hypothesis tests in the regression analysis, we applied the Holm-Bonferroni correction to control the family-wise error rate. Associations with adjusted *p*-values below the significance threshold were considered robust. Statistical significance was defined at *p* < 0.05.

## 3. Results

A total of 295 individuals were included in the study. The median age of the participants was 45.0 years (interquartile range, 32.5–58.0 years). Of the 295 patients, 54.9% were female and 45.1% were male. Diabetes mellitus (7.8%) was the most common comorbidity. The majority of individuals presented to the ED on the third (39.7%) day after the earthquake. CS occurred in 32.2% of participants, and RRT was required in 7.1% of cases.

According to baseline biochemical findings, the median duration of time spent under the rubble was 0 min (IQR: 0–60 min). Muscle injury markers were elevated, with a median CK level of 284.0 U/L (IQR: 122.0–894.0 U/L) and a median CK-MB level of 2.3 ng/mL (IQR: 1.3–4.4 ng/mL). Individuals with CS had significantly longer median duration of entrapment (120.0 min, IQR: 0.0–300.0) compared to those without (0.0 min, IQR: 0.0–0.0) (*p* < 0.001).

Individuals with CS had a significantly higher median heart rate (93.0 bpm vs. 88.0 bpm, *p* = 0.049). Markers of muscle injury, including CK and CK-MB, were markedly elevated in the CS group (CK: 1378.0 vs. 166.0 U/L, *p* < 0.001; CK-MB: 4.3 vs. 1.6 ng/mL, *p* < 0.001). Platelet counts were significantly lower in those with CS (248.5 ± 78.5 vs. 274.7 ± 77.6, *p* = 0.008), while pH levels were slightly but significantly higher (7.4 vs. 7.4, *p* = 0.012) (Table 1).

The need for RRT was considerably higher in individuals with CS, with 21.1% requiring RRT compared to 0.5% in those without (*p* < 0.001). Additionally, thoracic, lumbar, and neurovascular injuries were significantly more prevalent in the CS group, with thoracic injuries present in 13.7% (*p* = 0.003), lumbar injuries in 17.9% (*p* = 0.004), and neurovascular injuries in 4.2% (*p* = 0.010) (Table 1).

Multivariable logistic regression analysis is summarized in Table 2. CK was independently associated with CS, with an OR of 1.001 [95% CI: 1.001–1.002], which remained statistically significant after Holm–Bonferroni correction (adjusted *p* < 0.001). RRT showed an elevated OR of 13.472 (95% CI: 1.532–118.449), but its raw significance (*p* = 0.019) did not persist after adjustment for multiple testing. Duration under rubble was not an independent predictor (OR 1.002, 95% CI: 0.999–1.005; *p* = 0.162), despite its significance in univariable analysis.

In Table 3, a range of laboratory and clinical parameters were presented according to whether patients were trapped under rubble or not. Notably, those who were entrapped demonstrated higher median levels of respiratory rate, CK, and CK-MB (all *p* < 0.01). Although mean platelet counts were modestly lower in the entrapped group (*p* = 0.013), both subgroups exhibited values primarily within normal limits. No significant difference was observed in mean carbon dioxide levels (*p* = 0.813). Conversely, thoracic, abdominal, pelvic, and lumbar injuries were notably more frequent among those trapped (all *p* < 0.05).

## 4. Discussion

Depending on the circumstances, triaging algorithms can either over- or under-triage, despite their simplicity, straightforwardness, and ease of use [7]. The simple triage and rapid treatment (START) triage approach is the gold standard in Turkey for managing mass casualties, which involves assigning patients to triage colors based on their injury severity, and the first aid consists of a rapid assessment and quick movement of patients [14]. However, in this logic, patients triaged as green can be more seriously injured, yet both they and the healthcare providers are unaware of the severity.

Although it was not measured in the study, the hypothesis is that an interaction between the human body and the seismic waves produced in earthquakes may occur continuously, independent of whether they cause visible harm, and can determine injury patterns and consequences [15]. Due to ethical concerns, it is impossible to demonstrate this relationship on the human body; therefore, the adverse outcomes of this physical interaction in earthquake victims, whether or not there are signs of direct trauma, may be assessed through the presence of conditions such as CS and AKI, and the body’s behavior in response to seismic effects during an earthquake hypothesis, may be the merit of future studies. A significant number of patients in this study developed CS, despite being assigned to the green triage category (Table 1). Therefore, certain predictive markers, such as age, the nature and the mechanism of the injury, independent of the prejudice of being trapped, geographical location, and other characteristics of the affected individuals, should be prioritized and questioned by the healthcare teams before assigning patients to green triage to ensure proper functioning and prevent undertriage. There is a need for governments to investigate the behavior that led to mistakes following such major incidents. Furthermore, a standby resource deployment will enhance people’s access to resources in earthquake-prone areas of the country, particularly in rural areas, and prevent undertriaging [5].

As expected, the study was consistent with previous studies on the association of CS development and the risk factors [16]. However, some points should be emphasized in particular. First, while no independent correlation was found between the duration of entrapment and CS development (Table 2), which aligns with prior data [8], a correlation was found with CK levels. Although observed CK levels were much lower than expected, this can be attributed to most patients arriving after the 3rd day, as CK levels tend to decrease after this period [11]. In addition, even findings support that CK is the strongest independent predictor of CS; CK is a nonspecific marker that can be influenced by other covariates such as time since injury, hydration, metabolic factors, sex, age, muscle mass, physical activity, and race [17].

The second point to be emphasized here is the development of CS, traumatic injuries, and the need for RRT in those assigned to the green triage but not trapped, which points out that the human body can be affected by an earthquake in a clinically insidious manner, and this effect can be independent of whether a victim was trapped under the rubble or not (Table 1 and Table 3). A possible explanation for this may be that the great shaking force generated by an earthquake can cause a victim to lose their balance, fall to the ground, or even sustain severe injuries due to rapid and immense acceleration or vibration, which is similar to the damage produced by construction machinery. Although this does not represent the sole injury mechanism, it still provides a critical injury pathway for use in earthquake victims who stayed physically active in the aftermath of an earthquake, and triaged as green.

Considering the hypothesis and results of this study, every survivor is vulnerable to CS, regardless of the variables identified in the literature [18]. In disaster triage, the presence of an ambulatory victim suggests green and secondary triage [7]. As in other disasters, healthcare providers apply this triage during an earthquake. The patients included in the study stated that they did not feel injured due to the support of relatives during the search and rescue, confusion, or the adrenaline rush at the time of the incident, and therefore did not seek medical attention. However, those who did seek medical attention were evaluated under green patient triage. As experimental studies have demonstrated, exposure to seismic waves (including their intensity, proximity to the surface, and other factors) can lead to serious injuries [19]. The results of these experimental studies support the hypothesis regarding the possible injury mechanisms of green triage patients. Therefore, it is essential to revisit disaster triage. A multidisciplinary approach to implementing various public care systems or integrating interprofessional proposals offers benefits in disaster triage. Integrating the Diagnosis-Related Group (DRG) weight approach, which is quantified numerically by a standardized measure, determines the projected resource utilization based on the severity of a patient’s condition and the complexity of the interventions required, and the nursing care complexity, which ensures a nurse makes a diagnosis and takes action to encompass clinical practice strategies that meet healthcare demands, into the disaster triage can provide more accurate predictions of complications and resource demands in mass casualty events [20]. Establishing DRG weight and nursing complexity, implementing mobile health teams in government programs during and after disasters, and initiating treatment early as a result of repeated evaluations of victims who do not/cannot access health providers and receive a green code in the initial triage, can prevent morbidity and mortality, especially in patients assigned to the green triage.

This study presents several limitations. First, it was a single-center study that included patients presenting within 5 days and without prior medical attention. This can create a selection bias that limits generalizability for many earthquake victims who either seek care earlier/later or are treated in other facilities. Although selection bias can threaten the extrapolation of the results, the study population is representative of the target population, allowing for a focus on the clinical significance of reconsidering triage algorithms. Beyond this, study findings are context-specific (single hospital, one disaster, one triage system), which limits their generalizability to other disasters or healthcare systems. However, this limitation can be addressed in future studies by comparing the capability of triage systems in detecting serious injuries in patients assigned as green. Additionally, most patients presented on the third day, when CK levels are likely to be declining. This has resulted in some degree of timing bias, constituting an underestimation of the prevalence/severity of CS. On the other hand, despite this, a substantial proportion of patients have developed CS and received RRT. Furthermore, CK levels can be influenced by several conditions. This might create some degree of undercoverage bias. Also, this study has some statistical limitations. To explore potential predictors, multiple univariable analyses were followed by stepwise multivariable regression. Although this approach is commonly used in exploratory research, it may increase the risk of type I error and model instability. Therefore, the results should be interpreted with caution, and future confirmatory studies are encouraged to use theory-driven covariate selection or penalized regression methods (e.g., LASSO, ridge, or elastic net) to improve model robustness. Again, the large number of univariable comparisons performed increases the risk of type I error due to multiple testing. Although a formal correction for multiplicity in the primary analysis was not conducted, several marginal associations lost statistical significance under conservative Bonferroni adjustment. Notably, only the most robust findings—particularly the strong associations observed for CK, CK-MB, and related trauma severity indicators (all *p* < 0.001)—remained statistically significant, which supports the biological plausibility of the study results. Therefore, our conclusions are primarily based on these consistent and clinically meaningful associations rather than on borderline *p*-values. Consequently, the CIs for secondary and exploratory analyses have not been adjusted for multiple testing, and the findings should be interpreted cautiously as exploratory rather than confirmatory.

## 5. Conclusions

Reconsidering triage algorithms in mass casualties can be life-saving even in the absence of visible injuries. Even if the patient is ambulatory and assigned to green triage, a systematic evaluation, including CK monitoring, is necessary in this demographic to facilitate prompt identification and avert consequences such as AKI and the requirement for RRT.

## Figures and Tables

**Table 1:** Distribution of crush syndrome presence according to various demographic and biochemical values.

Variable	Absence of Crush Syndrome (n = 200)	Presence of Crush Syndrome (n = 95)	*p* Value
Age, median (IQR)	45.0 (32.0–59.0)	46.0 (33.0–57.0)	0.717 *
Gender, n (%)			
Female	116 (58.0)	46 (48.4)	0.156 **
Male	84 (42.0)	49 (51.6)	
Duration under the rubble, median (IQR)	0.0 (0.0–0.0)	120.0 (0.0–300.0)	<0.001 *
Pulse, median (IQR)	88.0 (81.8–96.2)	93.0 (85.0–98.5)	0.049
Body temperature, median (IQR)	36.5 (36.3–36.7)	36.4 (36.3–36.6)	0.303
Creatine kinase, median (IQR)	166.0 (90.4–352.0)	1378.0 (758.0–3364.0)	<0.001 *
Creatine kinase-MB, median (IQR)	1.6 (1.1–2.5)	4.3 (2.7–9.1)	<0.001 *
Platelets (PLT) (mean ± SD)	274.7 ± 77.6	248.5 ± 78.5	0.008 ***
pH, median (IQR)	7.4 (7.4–7.4)	7.4 (7.4–7.5)	0.012 *
CO_2_ (mean ± SD)	39.0 ± 5.6	38.8 ± 5.9	0.795 ***
Renal replacement therapy (RRT), n (%)			
No	199 (99.5)	75 (78.9)	<0.001 **
Yes	1 (0.5)	20 (21.1)	
Thorax injuries, n (%)			
No	176 (88.0)	71 (74.7)	0.005 **
Yes	23 (11.5)	24 (25.3)	
Thoracic vertebra involvement, n (%)			
No	193 (96.5)	82 (86.3)	0.003 **
Yes	7 (3.5)	13 (13.7)	
Lumbar vertebra involvement, n (%)			
No	187 (93.5)	77 (81.1)	0.004 **
Yes	13 (6.5)	17 (17.9)	
Neurovascular injury, n (%)			
No	200 (100.0)	90 (94.7)	0.010 ****
Yes	0 (0.0)	4 (4.2)	

* Mann–WhitneyMann-Whitney U test, ** chi-square test, *** Independent sample *t*-test, **** Fisher’s exact test.

**Table 2:** Multivariable logistic regression analyses of the association between crush syndrome and related factors.

Variable	OR [95% CI]	*p* Value
Duration of time under the rubble	1.002 [0.999–1.005]	0.162 *
Creatine kinase	1.001 [1.001–1.002]	<0.001 *
Renal replacement therapy (RRT)		
No (reference)	–	
Yes	13.472 [1.532–118.449]	0.019 *

The model was adjusted for age, sex, duration under rubble, pulse, creatine kinase, creatine kinase-MB, platelet count, renal replacement therapy, and injury site variables (thorax, thoracic, lumbar, neurovascular). OR: odds ratio; CI: confidence interval. OR: odds ratio, CI: confidence interval. * Multivariate analysis included variables with *p* < 0.10 in univariate analysis, and calculated by using the backward stepwise conditional method (10 steps). Statistically significant aftter Holm-Bonferroni indicated bold. Multiplicity was addressed by Holm–Bonferroni correction. Adjusted *p*-values are provided; only CK retained significance after correction. OR: odds ratio; CI: confidence interval.

**Table 3:** Distribution of various parameters according to being trapped under rubble.

Variables	Being Trapped		*p*
	No	Yes	
Respiratory rate (RR), median (IQR)	20 (18–20)	20 (18–21)	0.002 *
Creatine kinase (CK), median (IQR)	176 (96–475)	767 (215–2176)	<0.001 *
CK-MB, median (IQR)	1.8 (1.1–3.0)	3.3 (1.7–9.3)	<0.001 *
ISS trauma score, median (IQR)	0 (0–4)	4 (0–16.5)	<0.001 *
CO_2_ (mean ± SD)	38.9 ± 5.9	39.0 ± 4.8	0.813 **
Platelets (PLT) (mean ± SD)	273.8 ± 78.3	249.5 ± 76.3	0.013 **
Crush syndrome, n (%)			
No	152 (78.8)	29 (33.3)	<0.001 ***
Yes	41 (21.2)	58 (66.7)	
Head injury, n (%)			
No	178 (98.3)	96 (97.0)	0.669 ****
Yes	3 (1.7)	3 (3.0)	
Renal replacement therapy (RRT), n (%)			
No	176 (97.2)	84 (84.8)	<0.001 ***
Yes	5 (2.8)	15 (15.2)	
Neck injury, n (%)			
No	181 (97.2)	98 (99.0)	0.354 ****
Yes	0 (0.0)	1 (1.0)	
Maxillofacial trauma (MFT), n (%)			
No	178 (98.3)	97 (98.0)	0.999 ****
Yes	3 (1.7)	2 (2.0)	
Thorax injury, n (%)			
No	165 (91.2)	68 (69.4)	<0.001 ***
Yes	16 (8.8)	30 (30.6)	
Abdominal injury, n (%)			
No	179 (98.9)	93 (93.9)	0.025 ****
Yes	2 (1.1)	6 (6.1)	
Pelvic injury, n (%)			
No	179 (98.9)	92 (93.9)	0.024 ****
Yes	2 (1.1)	6 (6.1)	
Thoracic vertebra injury, n (%)			
No	175 (96.7)	86 (86.9)	0.003 ***
Yes	6 (3.3)	13 (13.1)	
Lumbar vertebra injury, n (%)			
No	170 (94.4)	80 (80.8)	<0.001 ***
Yes	10 (5.6)	19 (19.2)	
Neurovascular injury, n (%)			
No	181 (100.0)	94 (95.9)	0.015 ****
Yes	0 (0.0)	4 (4.1)	

* Mann–Whitney U test, ** Independent sample *t*-test, *** chi-square test, **** Fisher’s exact test.

## Data Availability

The raw data supporting the conclusions of this article will be made available by the authors on request.

## References

[1] Buyurgan Ç.S., Bozkurt Babuş S., Yarkaç A., Köse A., Usluer H.O., Ayrık C., Narcı H., Temel G. (2023). Demographic and Clinical Characteristics of Earthquake Victims Presented to the Emergency Department with and without Crush Injury upon the 2023 Kahramanmaraş (Turkey) Earthquake. Prehosp. Disaster Med..

[2] Comoglu M., Acehan F., Inan O., Demir B.F., Yılmaz Y., Sahiner S.E. (2025). A new score predicting renal replacement therapy in patients with crush injuries: Analysis of a major earthquake. Am. J. Emerg. Med..

[3] Earthquake Hazards Program. https://www.usgs.gov/programs/earthquake-hazards/earthquake-magnitude-energy-release-and-shaking-intensity.

[4] Bazyar J., Farrokhi M., Khankeh H. (2019). Triage Systems in Mass Casualty Incidents and Disasters: A Review Study with a Worldwide Approach. Open Access Maced. J. Med. Sci..

[5] Bazyar J., Farrokhi M., Salari A., Safarpour H., Khankeh H.R. (2022). Accuracy of Triage Systems in Disasters and Mass Casualty Incidents; a Systematic Review. Arch. Acad. Emerg. Med..

[6] Wang J., Lu W., Hu J., Xi W., Xu J., Wang Z., Zhang Y. (2022). The Usage of Triage Systems in Mass Casualty Incident of Developed Countries. Open J. Emerg. Med..

[7] Clarkson L., Williams M. (2025). EMS Mass Casualty Triage. StatPearls [Internet].

[8] Long B., Liang S.Y., Gottlieb M. (2023). Crush injury and syndrome: A review for emergency clinicians. Am. J. Emerg. Med..

[9] Nola I.A., Dvorski M., Žižak M., Kuliš T. (2023). Crush syndrome in earthquakes—Stay and play or load and go?. Acta Clin. Croat..

[10] Abdul-Nabi S.S., Hitti E. (2025). A review of mass casualty incident triage tools for hospital-based triage. Turk. J. Emerg. Med..

[11] Gur A., Simsek Y. (2024). The impact of creatine kinase and base excess on the clinical outcome of crush injuries sustained during the Kahramanmaras/Turkey earthquakes on February 6, 2023. Medicine.

[12] Haines L.N., Doucet J.J. Severe Crush Injury in Adults. https://www.uptodate.com.

[13] Fernandez J.J., Smith S.R. (2025). Traumatic Rhabdomyolysis: Crush Syndrome, Compartment Syndrome, and the ‘Found Down’ Patient. J. Am. Acad. Orthop. Surg..

[14] Wisnesky U.D., Kirkland S.W., Rowe B.H., Campbell S., Franc J.M. (2022). A Qualitative Assessment of Studies Evaluating the Classification Accuracy of Personnel Using START in Disaster Triage: A Scoping Review. Front. Public Health.

[15] Kai Y., Kajiwara K. (2011). Study on dynamic response characteristics of human body under the seismic motion using shaking table test of crash test dummies. AIJ J. Technol. Des..

[16] Turgutalp K., Kıykım A.A., Oto Ö.A., Demir S., Çobanoğlu D., Oğuz İ., Ünver R.E., Akın Şen İ., Özer C., Sever M.Ş. (2024). Analysis of Crush Syndrome Patients With and Without Acute Kidney Injury during the 2023 Kahramanmaraş Earthquake: Experience of a Tertiary Referral Center from Türkiye. Turk. J. Nephrol..

[17] Aujla R.S., Zubair M., Patel R. (2025). Creatine Phosphokinase. StatPearls [Internet] 2024.

[18] Akrivos V.S., Koutalos A., Stefanou N., Koskiniotis A., Arnaoutoglou C. (2025). Crush injury and crush syndrome: A comprehensive review. EFORT Open Rev..

[19] Cimellaro G.P., Domaneschi M., Boroschek R., Cardoni A., Kammouh O., Galdo D., Apostoliti C., Cares D., Mahin S. Monitoring human body motions during earthquakes. Proceedings of the 11th National Conference in Earthquake Engineering, Earthquake Engineering Research Institute.

[20] Cesare M., D’Agostino F., Sebastiani E., Damiani G., Cocchieri A., Nursing and Public Health Group (2025). Deciphering the Link Between Diagnosis-Related Group Weight and Nursing Care Complexity in Hospitalized Children: An Observational Study. Children.

